# Revisiting the Role of Radiation Therapy in Chondrosarcoma: A National Cancer Database Study

**DOI:** 10.1155/2019/4878512

**Published:** 2019-10-13

**Authors:** Anthony A. Catanzano, David L. Kerr, Alexander L. Lazarides, Brian L. Dial, Whitney O. Lane, Dan G. Blazer, Nicole A. Larrier, David G. Kirsch, Brian E. Brigman, William C. Eward

**Affiliations:** ^1^Department of Orthopaedic Surgery, Duke University Medical Center, 2301 Erwin Rd, Durham, NC 27710, USA; ^2^Department of General Surgery, Duke University Medical Center, 2301 Erwin Rd, Durham, NC 27710, USA; ^3^Department of Radiation Oncology, Duke University Medical Center, 2301 Erwin Rd, Durham, NC 27710, USA

## Abstract

**Background:**

Although chondrosarcomas (CS) are mostly considered radioresistant, advancements in radiotherapy have brought attention to its use in these patients. Using the largest registry of primary bone tumors, the National Cancer Database (NCDB), we sought to better characterize the current use of radiotherapy in CS patients and identify any potential survival benefit with higher radiation doses and advanced radiation therapies.

**Methods:**

We retrospectively analyzed CS patients in the NCDB from 2004 to 2015 who underwent radiotherapy. The Kaplan–Meier method with statistical comparisons was used to identify which individual variables related to dosage and delivery modality were associated with improved 5-year survival rates. Multivariate proportional hazards analyses were performed to determine independent predictors of survival.

**Results:**

Of 5,427 patients with a histologic diagnosis of chondrosarcoma, 680 received a form of radiation therapy (13%). The multivariate proportional hazards analysis controlling for various patient, tumor, and treatment variables, including RT dose and modality, demonstrated that while overall radiation therapy (RT) was not associated with improved survival (HR 0.96, 95% CI 0.76–1.20), when examining just the patient cohort with positive surgical margins, RT trended towards improved survival (HR 0.81, 95% CI 0.58–1.13). When comparing advanced and conventional RT modalities, advanced RT was associated with significantly decreased mortality (HR 0.55, 95% CI 0.38–0.80). However, advanced modality and high-dose RT both trended only toward improved survival compared to patients who did not receive any RT (HR 0.74, 95% CI 0.52–1.06 and HR 0.93, 95% CI 0.71–1.21, respectively).

**Conclusions:**

Despite the suggested radioresistance of CS, modern radiotherapies may present a treatment option for certain patients. Our results support a role for high-dose, advanced radiation therapies in selected high-risk CS patients with tumors in surgically challenging locations or unplanned positive margins. While there is an associated survival rate benefit, further, prospective studies are needed for validation.

## 1. Introduction

Chondrosarcoma is the second most common primary bone malignancy in the United States, representing approximately 2,000 new cases every year [1]. Most commonly, tumors are located within the appendicular skeleton and pelvis; however, recent data report up to 15% occur within the vertebral column [2]. Negative prognostic indicators for survival include tumor grade, patient age, axial tumor location, local recurrence, and inadequate surgical resection [3]. While chondrosarcomas within the appendicular skeleton are often amenable to limb salvage treatment with negative margin resection, those within the vertebral column and pelvis are more challenging to resect because of the surrounding anatomical structures [4]. For these reasons, adjuvant chemotherapy and radiation therapy may be useful tools in the treatment of chondrosarcomas.

Despite being classified by some clinicians as resistant to chemotherapy and radiation therapy, one retrospective series from Princess Margaret Hospital suggested that the addition of standard doses (50 Gy) of radiation therapy to surgery leads to improved outcomes [5, 6]. Whereas higher doses of radiation may be even more efficacious, the dose of radiation delivered to tumors located in the axial skeleton is restricted by the spinal cord, nearby neurovascular structures and bowel, which further limits its use in CS. Advances in radiation therapy technology have allowed for higher radiation doses to be delivered with sharp dose gradients, providing a more potent dose directly to tumor tissue while sparing surrounding normal tissues.

Given the low incidence of these tumors and the presumption of radioresistance by some clinicians, there are limited studies of large cohorts to assess the effect of radiotherapy on survival outcomes in chondrosarcoma patients. Using the largest registry of primary bone tumors, the National Cancer Database (NCDB), we sought to characterize the use of radiotherapy in CS patients. The NCDB is the most complete tumor registry available, capturing 70% of all newly diagnosed cancers in the United States [7, 8]. In addition, the NCDB provides more complete treatment and patient data compared to other registries, such as the SEER registry, in regard to nonsurgical modalities such as radiotherapy and systemic chemotherapy by including data on surgical margin status, socioeconomic data, and survival outcomes [8]. A previous investigation of CS in the NCDB has been performed; however, this study was limited to the head and neck [9].

## 2. Methods

The institutional review board of our institution approved this retrospective analysis of the NCDB for patients diagnosed with chondrosarcomas from 2004 to 2015. The NCDB Participant User File was searched for patients treated at NCDB-participating institutions with a primary histologic diagnosis of chondrosarcoma, treated with radiotherapy, with a reported dose and delivery modality. Radiation modalities included conventional EBRT as well as intensity-modulated radiation therapy (IMRT), proton-beam therapy (PBT), and stereotactic radiosurgery (SRS), which were all categorized as advanced modalities, given a more precise delivery of radiation to tumor tissue. The patients were identified using the International Classification of Diseases for Oncology, 3rd Edition (ICD-O-3) topography codes C41.0 (bones and joints: skull, face bones), C41.2 (bones and joints: vertebral column), C41.4 (bones and joints: pelvis, sacrum, coccyx), and C40.0, C40.1, C40.2, C40.3, C40.8, and C40.9 (all bones, joints, and articular cartilage of limbs).

Data from the NCDB were available to use from 2004 to 2015. Exclusion criteria included patients that did not have a primary chondrosarcoma, patients with mesenchymal chondrosarcoma, patients with additional secondary malignancies (sequence number >1), and patients for whom it was unknown if they received beam radiation therapy. From the NCDB dataset, 5,427 chondrosarcomas were identified meeting criteria of which 680 were treated with radiotherapy at the reporting facilities between 2004 and 2015 and met our study criteria.

The chondrosarcoma patients were first divided by radiation modality (conventional (EBRT) vs. advanced (IMRT, PBTE, and SRS)). Patient characteristics, tumor characteristics, and treatment characteristics were compared between the different treatment groups. The following variables were compared: (1) patient characteristic variables: age, sex, race, Charlson Comorbidity Score (CCS), income (based on the average income level in the zip code of the patient's home), facility type (academic/research program or community cancer program), and insurance status (none, private insurance, and government insurance including Medicare and Medicaid); (2) tumor characteristic variables: tumor size, tumor grade, and tumor site; and (3) treatment variables: surgical resection, surgical margin status, type of radiotherapy (as mentioned above), and radiation dose (<40, 40–60, and >60 Gy). In patients receiving less than 40 Gy of radiation, it was inferred this treatment was given with a palliative, rather than curative intent. The other groups were considered high-dose (>60 Gy) and low-dose (40–60 Gy) groups if the radiation modality was a type other than stereotactic radiosurgery (SRS), as this group receives lower cumulative doses in higher individual-dose fractions.

### 2.1. Statistical Analysis

Demographic, clinical, and outcome data were compiled and presented utilizing descriptive statistics. Patient cohorts were identified by whether or not they received radiation, whether the radiation modality was conventional or advanced, and by whether the radiation dose the patient received was considered high-, low-, or palliative. Patient groups with different radiotherapy modalities for chondrosarcoma were assessed for differences in patient, tumor, and treatment characteristics using Fisher's exact and Pearson's chi-square tests for categorical variables and two-tailed *t*-tests or ANOVA tests for continuous variables such as patient age. Patient, tumor, and treatment variables to be included in the multivariate Cox proportional hazards model were assessed in univariate Kaplan–Meier analysis. Five-year survival estimates were obtained from Kaplan–Meier curves while stratifying across treatment type. Survival comparisons between radiation modalities were also assessed for interaction with surgical margin status both via the Kaplan–Meier analysis and the Cox proportional hazards model.

Multivariate proportional hazards analysis was used to identify patient, tumor, and treatment characteristics associated with increased mortality. Multiple imputation for missing data included all patient, tumor, and treatment variables examined in the analysis, as well as survival time and censoring data. Twenty imputations were performed and used in proportional hazards regression. Variables included in the base model were age (above or below median of 53 years), sex, race, Charlson Comorbidity Score, insurance type (private, government, or none), income (above or below median), facility type (academic or community), tumor size, grade, metastases at diagnosis, surgery and margin status, radiation type, and chemotherapy. Radiation types were grouped by both modality (advanced or conventional) and dose (high, low, or palliative). In a second model, the analysis was repeated including an interaction term between radiation and margin status, in which patients with positive margin status who received radiation therapy were compared to those with positive margins who did not receive adjuvant radiation. In a third model, radiation modality groups compared EBRT and IMRT together against PBT. Hazard ratios and 95% confidence intervals were computed for all covariates, with *p* values <0.05 indicating statistical significance. All statistical analyses were performed using SAS/JMP (SAS Institute Inc, Cary, NC, USA).

## 3. Results

A total of 5,427 patients with a histologic diagnosis of chondrosarcoma were identified. 52% were located in the appendicular skeleton, while 18% were within the pelvis and 4% in the spine, consistent with the previously published data. Of all the identified CS patients, 680 received some form of radiation therapy (13%).

Table 1 compares patient characteristics between CS patients receiving radiation therapy (RT) and CS patients not treated with radiation therapy (no RT), while Table 2 summarizes tumor and treatment characteristics between the two groups. Those receiving RT were overall similar to the cohort not receiving RT in regard to gender, race, comorbidities, education, and income level, as well as insurance status. When paralleling tumor characteristics, those receiving RT were more likely to have a primary location of the head, neck, or spine. Only 22% of the RT-treated patients have upper or lower extremity CS, while extremity tumors make up 56% of all those within the NCDB with CS who did not receive RT. When compared to the general CS cohort, those receiving RT were more likely to be of high or intermediate grade (73% vs. 55%, *p* < 0.001), and more than twice as many patients who received RT were also undergoing chemotherapy (14% vs. 5%). The RT cohort had significantly higher rates of positive margins after surgical resection (44% vs. 12%, *p* < 0.001).

**Figure 1:**
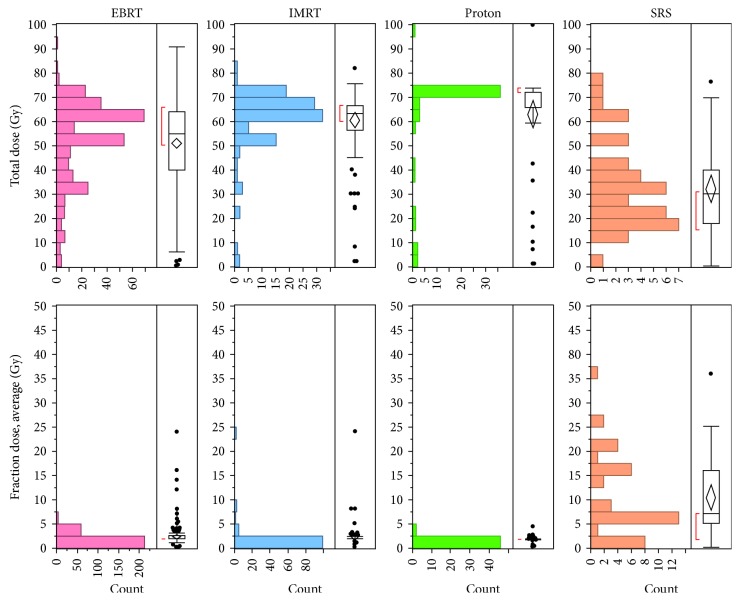
Distributions for total radiation doses (top row) as well as fraction doses (bottom row) for each radiation modality. Stereotactic radiosurgery (SRS) is typically given as high-dose fractures for a lower total dose.

Tables 1 and 2 also highlight the type of radiation therapy CS patients have received. 294 patients (55%) received conventional EBRT and 245 (45%) patients received advanced radiation therapy modalities, including IMRT, PBT, and SRS. Overall, 42% of patients treated with a non-SRS modality received high-dose therapy (>60 Gy). The median total and fractional doses of SRS were 30 and 7 Gy, compared to 60 and 2 Gy for the other modalities (Figure 1). Few significant differences in demographics were found amongst patient characteristics between high- and low-dose RT groups or between conventional and advanced modality groups, with the exceptions of patient age and treatment facility type. Patients receiving conventional EBRT or low- or palliative-dose RT were more likely to be older or treated at nonacademic facilities. Stratifying by tumor site, tumors of the head and neck most commonly received both high-dose and advanced therapies, followed by tumors of the spine.

**Figure 2:**
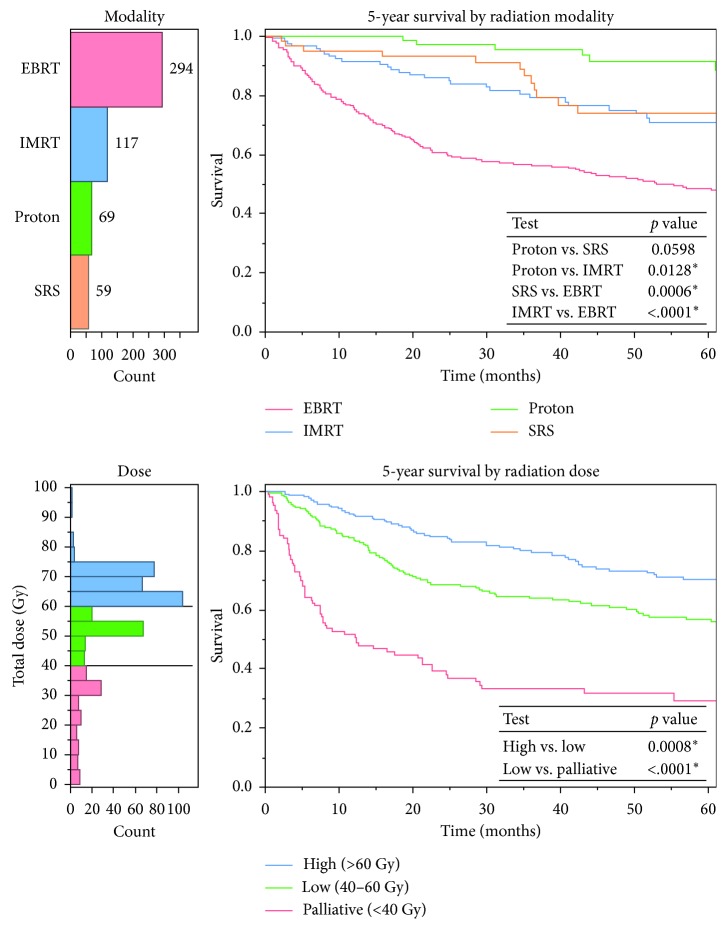
Kaplan–Meier curves comparing survival between radiation treatment groups, stratified by (a) modality and (b) dose. Modalities included EBRT, IMRT, PBT, and SRS. Dose groups included high-dose (>60 Gy), low-dose (40–60 Gy), and palliative (<40 Gy). Modality and dose distributions shown to the left of survival curves. Log-rank tests comparing treatment groups included in KM curve inset, with alpha = 0.05.

**Figure 3:**
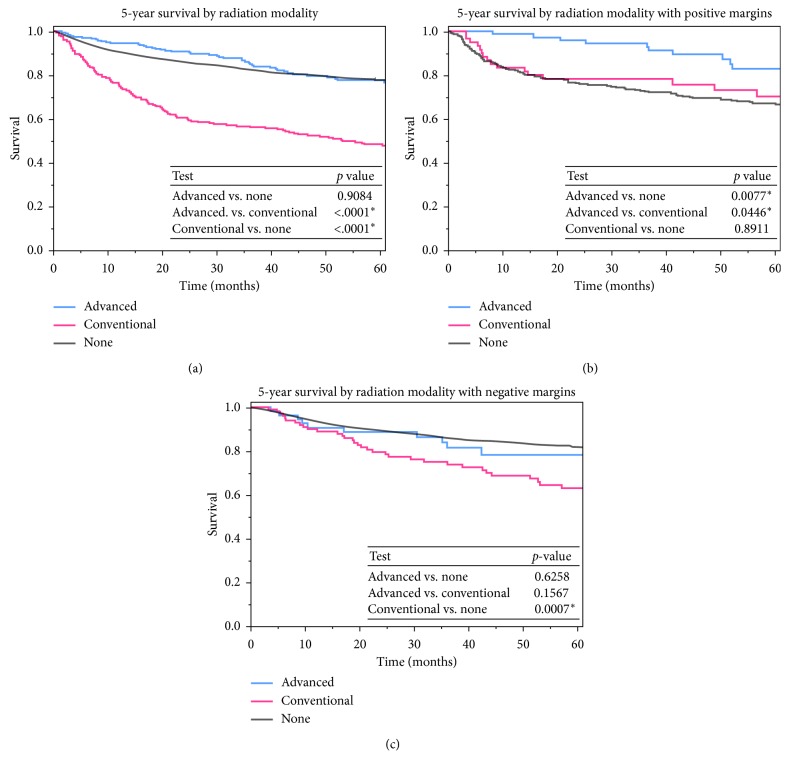
Kaplan–Meier curves comparing survival for patients who received no RT and those who received conventional RT (EBRT) or advanced modality RT (IMRT, PBT, and SRS). (a) Overall 5-year survival rates were compared, as well as 5-year survival rates in patient groups with (b) positive or (c) negative surgical margins after resection.

The 5-year survival rates of those receiving high-dose (>60 Gy) RT were 70%, significantly higher than the 57% survival rate of low-dose (40–60 Gy) RT (*p* < 0.001, Figure 2). When comparing conventional EBRT to advanced RT modalities, regardless of dose, there was a significant improvement in overall 5-year survival rates of advanced modalities (combined 78% vs. conventional 48%, *p* < 0.001, Figure 3). The overall survival rate of those patients receiving advanced modality RT was similar to those who did not receive any RT. However, after controlling for positive margin status in patients after surgical resection, patients who received advanced modality RT had significantly improved survival with positive margin surgery than patients who did not receive RT (*p*=0.0077, Figure 3).

**Figure 4:**
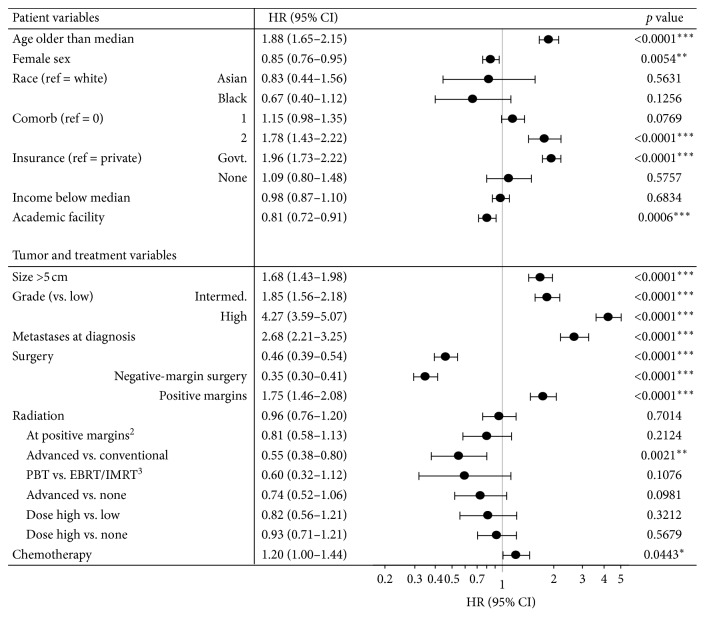
Multivariate Cox proportional hazards regression for independent predictors of mortality in patients with chondrosarcoma. Forest plot of hazard ratios and 95% confidence intervals displayed for patient, tumor, and treatment variables. Base model performed with radiation type variable categorized by both radiation modality and dose. ^2^Model repeated to assess the impact of margin status on the survival effect of radiation therapy. ^3^Radiation modalities of EBRT and IMRT were combined and compared with PBT.

In a multivariate proportional hazards analysis controlling for various patient, tumor, and treatment variables, older age, male sex, comorbidity score >1, government insurance rather than private, nonacademic facility type, larger tumor size, higher tumor grade, lack of surgery, or positive margin surgery were all associated with worse survival. While overall RT and chemotherapy were not associated with improved survival (HR 0.96, 95% CI 0.76–1.20 for RT; HR 1.20, 95% CI 1.00–1.44 for chemotherapy), when examining just the patient cohort with positive margins, RT overall trended towards improved survival (HR 0.81, 95% CI 0.58–1.13). Compared to advanced modality RT, conventional RT was associated with significantly increased mortality, though advanced modality and high-dose RT both trended only toward improved survival compared to patients who did not receive any RT (Figure 4).

## 4. Discussion

As demonstrated by our review of the NCDB, despite the suggested radioresistance of chondrosarcoma, radiotherapy should be strongly considered for these patients, especially those with tumors located in difficult areas for radical resection, such as the skull base, spine, and pelvis, which are anatomic sites with a high percentage of positive margins after resection. While surgical resection should be performed with the intent of obtaining negative margins, there remains the risk of compromised function and significant morbidity when operating in these anatomic areas. Our results support a role for radiation therapy in selected high-risk CS patients with tumors in surgically challenging locations or for unplanned positive margins. Furthermore, the utilization of advanced modalities with radiation dose greater than 60 Gy in these patients is associated with an overall survival benefit.

Wide resection to achieve negative margins remains the gold standard treatment of chondrosarcoma to maximize overall patient survival and limit local recurrence [10]. Consistent with other reported studies and our analysis of the NCDB, obtaining negative margins of CS through radical resection correlates to longer survival rates, regardless of adjuvant therapies. This has been established as a critical prognostic factor in CS of the pelvis, sacrum, and vertebral column [11, 12]. Obtaining negative margins becomes challenging with these tumors located in the axial skeleton and pelvis. The surrounding bowel, bladder, and iliac vessels must be considered in the resection of pelvic tumors, while the spinal cord and nerve roots present limitations in resection of vertebral column tumors [3, 13]. In these clinical settings, effective adjuvant therapy has the potential to improve outcome for CS.

While there is an established benefit of adjuvant radiation therapy on local recurrence of wide resection of limb soft tissue sarcomas with negative margins, Delaney et al. reported its use specifically in cases with positive margins, demonstrating improved survival and lower recurrence rates for those treated with radiation therapy [14–16]. Other studies have also suggested benefits of decreased local recurrence from adjuvant radiation therapy in sarcoma cases with positive margins and tumors surrounding critical anatomical structures [17]. Although many of these early studies focused mainly on soft tissue sarcomas, recent studies report similar, encouraging results when used for primary sarcomas of bone, such as osteosarcoma and Ewing sarcoma [18–21].

The majority of reports of radiation therapy for chondrosarcoma have focused on skull base tumors. Given the proximity of skull base tumors to the brainstem and other cranial structures, achieving negative margins can be difficult, and the need for precise adjuvant radiation therapy is essential. As validated by our review of the NCDB, head and skull base tumors are the most common CS to receive RT. Several studies have reported positive results of adjuvant radiotherapy in skull base CS, despite its reported radioresistance [22, 23]. Few studies have evaluated its use in extracranial, nonskull base CS. One such study of Goda et al. reported excellent local recurrence rates and 10-year overall and progression-free survival rates on 60 extracranial chondrosarcoma patients who underwent surgical resection and radiation therapy, including 13 patients with pelvic tumors [5]. This represents a small cohort and other than radiation dose, the specific radiation modalities utilized in these patients were not reported. However, this case series provides justification for further investigations into the use of RT as an adjuvant therapy in patients with extracranial CS, especially axial tumors where it may be most beneficial.

In cases where residual tumor remains after resection given proximity to vital structures, IMRT and/or proton therapy may provide adjuvant therapy that improves survival and local recurrence rates. Recent literature has begun to investigate the use of radiation therapy in these settings; however, patient cohorts remain small given the rarity of these tumors [24]. DeLaney et al. have reported a phase II study of high-dose photon/proton therapy used before and after surgical resection in patients with CS. Their results of 14 CS patients show 5-year local control rates of 78% and a survival rate of 87%, both improved from the earlier reported means [25].

Through our review of the NCDB, when treated with RT, patients with positive margins have improved survival rates when advanced modalities, such as IMRT or PBT, are utilized, with a trend towards improved survival with higher radiation doses. This highlights the importance for patients to be evaluated by radiation oncologists with experience treating sarcomas to consider the potential use of advanced modalities and higher radiation doses to sterilize tumor margins while preserving critical structures to minimize the risk of toxicity.

Although the results of our investigation are encouraging, they are not without limitations. The NCDB does not report local recurrence rates. As survival of these patients depends on metastases and other patient variables, local recurrence may provide a more straightforward indicator of the success of local adjuvant therapy, as the direct impact of radiation therapy on overall survival is difficult to surmise. Other important factors not considered in this analysis include reoperation and rehospitalization rate, as well as quality of life and functional scores. Assessing our treatment groups, although SRS is identified as an advanced modality, it is difficult to interpret the effect stratified by dose. SRS is given with a high daily radiation dose; however, the total overall dose is less than either IMRT or PBT and for this reason was excluded from the dosage analysis. In addition, for the cohort of patients with positive margins, microscopic vs. gross residual disease is not specified. This may lead to patients classified within the same cohort (positive surgical margins) having different baseline prognoses and potentially skew the survival rates.

Furthermore, the NCDB dataset is not complete for all patients included in our analyses. Patients without specific data for variables were not included in those comparisons. Given the retrospective nature of our study, many of our conclusions are inferred from the collected data. These inferences provide starting points for future studies; however, in order to truly assess the usefulness of radiation therapy in chondrosarcoma, prospective studies of larger cohorts are needed to validate these findings and investigate local recurrence rates.

## 5. Conclusions

Given the difficulty of obtaining negative margins in CS of the pelvis and axial skeleton, effective adjuvant therapies are imperative to improving outcomes and survival rates. With advances of radiation therapy allowing improved precision of higher radiation doses adjacent to normal critical structures, our results correlating improved survival with RT suggest that it may be a useful adjuvant modality in selected CS patients with positive margins. Successful use of these modalities may not only preserve critical structures but also allow for less morbid resections and improved limb salvage options. Larger, prospective studies focusing on advanced modalities of RT with high dose for CS of the axial skeleton and pelvis are needed to better define the role of RT in these patients.

## Figures and Tables

**Table 1:** Patient characteristics.

*N* (%)		No radiation (*n* = 4742)	Radiation (*n* = 680)	High dose >60 Gy (*n* = 228)	Low dose 40–60 Gy (*n* = 204)	Palliative <40 Gy (*n* = 107)	Conventional (*n* = 294)	Advanced (*n* = 245)
Age, mean (SD) in years		52 (52-53)^*∗∗∗*^	55 (53–56)^*∗∗∗*^	52 (50–54)^*∗∗∗*^	58 (55–60)^*∗∗∗*^	60 (56–63)^*∗∗∗*^	58 (56–60)^*∗∗∗*^	50 (48–53)^*∗∗∗*^

Gender	Female	2272 (48)	308 (45)	102 (45)	91 (45)	52 (49)	139 (47)	110 (45)

Race	Asian	116 (3)	18 (3)	4 (2)	6 (3)	3 (3)	6 (2)	8 (3)
	Black	319 (7)	49 (7)	10 (4)	15 (7)	12 (11)	21 (7)	14 (6)
	White	4129 (89)	595 (89)	209 (92)	179 (89)	89 (85)	262 (90)	215 (90)

Hispanic ethnicity		343 (8)^*∗*^	67 (10)^*∗*^	27 (12)	17 (9)	10 (10)	27 (10)	26 (11)

Comorbidity	0	3964 (84)	559 (82)	183 (80)	165 (81)	91 (85)	235 (80)	212 (87)
	1	616 (13)	92 (14)	37 (16)	28 (14)	12 (11)	48 (16)	24 (10)
	>1	167 (4)	29 (4)	8 (4)	11 (5)	4 (4)	11 (4)	9 (4)

Insurance	Private	2777 (61)^*∗∗*^	361 (54)^*∗∗*^	122 (54)	107 (53)	47 (44)	140 (48)	150 (62)
	Government	1564 (34)^*∗∗*^	276 (41)^*∗∗*^	87 (39)	85 (42)	55 (51)	130 (45)	84 (35)
	None	220 (5)^*∗∗*^	35 (5)^*∗∗*^	17 (8)	9 (4)	5 (5)	21 (7)	9 (4)

Income above median		2791 (60)	416 (62)	142 (62)	119 (59)	64 (60)	173 (59)	157 (64)

Academic facility type		2375 (66)^*∗∗∗*^	281 (54)^*∗∗∗*^	100 (60)^*∗*^	74 (44)^*∗*^	50 (56)^*∗*^	114 (47)^*∗∗∗*^	107 (64)^*∗∗∗*^

For each value, the (%) reflects only the proportion of patients with known values for each variable (unknown values not included in %). Statistical comparisons included RT vs. none, palliative vs. low vs. high doses (non-SRS modalities), and conventional vs. advanced modalities. Fisher's exact or Pearson chi-square tests were performed for categorical variables. Two-tailed t-test or ANOVA performed for numerical variables (age). Statistical significance indicated by ^*∗*^ for *p* < 0.05, ^*∗∗*^ for *p* < 0.01, and ^*∗∗∗*^ for *p* < 0.001.

**Table 2:** Tumor and treatment characteristics.

*N* (%)		No radiation (*n* = 4742)	Radiation (*n* = 680)	High dose >60 Gy (*n* = 228)	Low dose 40–60 Gy (*n* = 204)	Palliative <40 Gy (*n* = 107)	Conventional (*n* = 294)	Advanced (*n* = 245)
Tumor size ≥ 5 cm		2628 (66)^*∗*^	325 (61)^*∗*^	100 (53)^*∗∗∗*^	122 (75)^*∗∗∗*^	59 (76)^*∗∗∗*^	168 (71)^*∗∗∗*^	86 (45)^*∗∗∗*^

Tumor site	Axial	2012 (44)^*∗∗∗*^	507 (78)^*∗∗∗*^	180 (83)^*∗∗∗*^	141 (73)^*∗∗∗*^	63 (64)^*∗∗∗*^	190 (69)^*∗∗∗*^	221 (92)^*∗∗∗*^
	Appendicular	2600 (56)^*∗∗∗*^	140 (22)^*∗∗∗*^	37 (17)^*∗∗∗*^	53 (27)^*∗∗∗*^	36 (36)^*∗∗∗*^	84 (31)^*∗∗∗*^	20 (8)^*∗∗∗*^

Grade	Low	1868 (45)^*∗∗∗*^	144 (27)^*∗∗∗*^	57 (30)^*∗∗*^	36 (24)^*∗∗*^	11 (13)^*∗∗*^	48 (21)^*∗∗∗*^	69 (35)^*∗∗∗*^
	Intermediate	1541 (37)^*∗∗∗*^	235 (45)^*∗∗∗*^	90 (47)^*∗∗*^	68 (46)^*∗∗*^	36 (44)^*∗∗*^	102 (45)^*∗∗∗*^	94 (48)^*∗∗∗*^
	High	773 (18)^*∗∗∗*^	146 (28)^*∗∗∗*^	43 (23)^*∗∗*^	44 (30)^*∗∗*^	35 (43)^*∗∗*^	79 (35)^*∗∗∗*^	34 (17)^*∗∗∗*^

Metastases at diagnosis		191 (4)^*∗∗∗*^	73 (11)^*∗∗∗*^	7 (3)^*∗∗∗*^	18 (9)^*∗∗∗*^	43 (42)^*∗∗∗*^	48 (17)^*∗∗∗*^	9 (4)^*∗∗∗*^

Surgery		4328 (91)^*∗∗∗*^	513 (75)^*∗∗∗*^	190 (83)^*∗∗∗*^	152 (75)^*∗∗∗*^	56 (52)^*∗∗∗*^	201 (68)^*∗∗∗*^	199 (81)^*∗∗∗*^

Margin	Positive	415 (12)^*∗∗∗*^	168 (44)^*∗∗∗*^	68 (49)^*∗*^	42 (34)^*∗*^	15 (38)^*∗*^	60 (38)^*∗∗∗*^	80 (60)^*∗∗∗*^

Chemotherapy		249 (5)^*∗∗∗*^	91 (14)^*∗∗∗*^	19 (9)^*∗∗∗*^	27 (14)^*∗∗∗*^	28 (26)^*∗∗∗*^	48 (17)^*∗∗*^	18 (8)^*∗∗*^

For each value, the (%) reflects only the proportion of patients with known values for each variable (unknown values not included in %). Statistical comparisons included RT vs. none, palliative vs. low vs. high doses (non-SRS modalities), and conventional vs. advanced modalities. Fisher's exact or Pearson chi-square tests were performed for categorical variables. Two-tailed t-test or ANOVA was performed for numerical variables (age). Statistical significance indicated by ^*∗*^ for *p* < 0.05, ^*∗∗*^ for *p* < 0.01, and ^*∗∗∗*^ for *p* < 0.001.

## Data Availability

The patient data used to support the findings of this study may be released upon application to the National Cancer Database Participant User File, who can be contacted at https://www.facs.org/quality-programs/cancer/ncdb/puf.
